# Supplementary material for: High-Temperature Magnesiothermic Reduction Enables HF-Free Synthesis of Porous Silicon with Enhanced Performance as Lithium-Ion Battery Anode
Source: Molecules. 2022 Nov 2;27(21):7486. doi: 10.3390/molecules27217486 (PMC9655285; doi:10.3390/molecules27217486)
Supplement: Supplementary file 1 [file molecules-27-07486-s001.zip › molecules-1972331-supplementary.pdf]

High-Temperature Magnesiothermic Reduction Enables HF-Free Synthesis of Porous Silicon with Enhanced Performance as Lithium-Ion Battery Anode

Xiuxia Zuo[1], Qinghua Yang[2], Yaolong He[2*], Ya-Jun Cheng[1, 3*], Shanshan Yin[4], Jin Zhu[1], Peter Müller-Buschbaum[4, 5], and Yonggao Xia[1, 6*]

1. Ningbo Institute of Materials Technology & Engineering, Chinese Academy of Sciences, 1219 Zhongguan West Rd, Ningbo, 315201, Zhejiang Province, P. R. China
2. Shanghai Institute of Applied Mathematics and Mechanics, School of Mechanics and Engineering Science, Shanghai University, Shanghai, 200444, China;
3. Department of Materials, University of Oxford, Parks Rd, OX1 3PH, Oxford, UK
4. Physik-Department, Lehrstuhlfür Funtionelle Materielien, Technische Universität München, James-Franck-Strasse 1, 85748 Garching, Germany
5. Heinz Maier-Leibnitz Zentrum (MLZ), Technische Universität München, Lichtenbergstr. 1, 85748 Garching, Germany
6. Center of Materials Science and Optoelectronics Engineering, University of Chinese Academy of Sciences, 19A Yuquan Rd, Shijingshan District, Beijing 100049, P. R. China

Email address: yaolonghe@shu.edu.cn, chengyj@nimte.ac.cn, xiayg@nimte.ac.cn

Supplementary Material

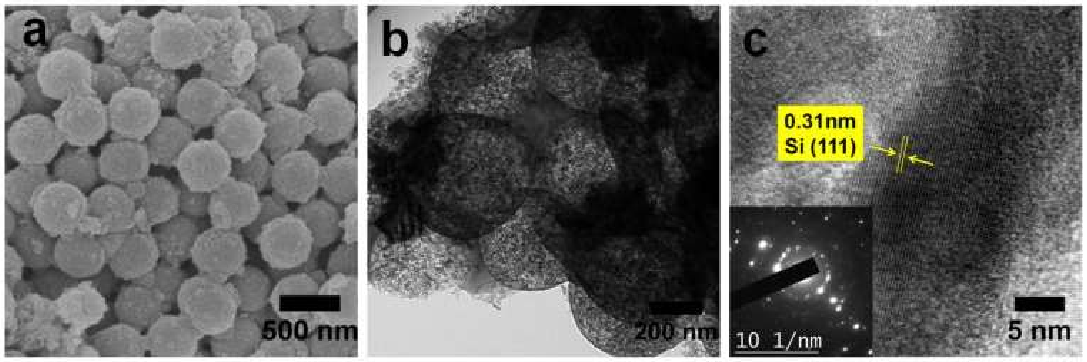

Figure S1. SEM (a), TEM (b), HRTEM (c) images and SAED pattern (inset of image c) of the 700-HCl sample.

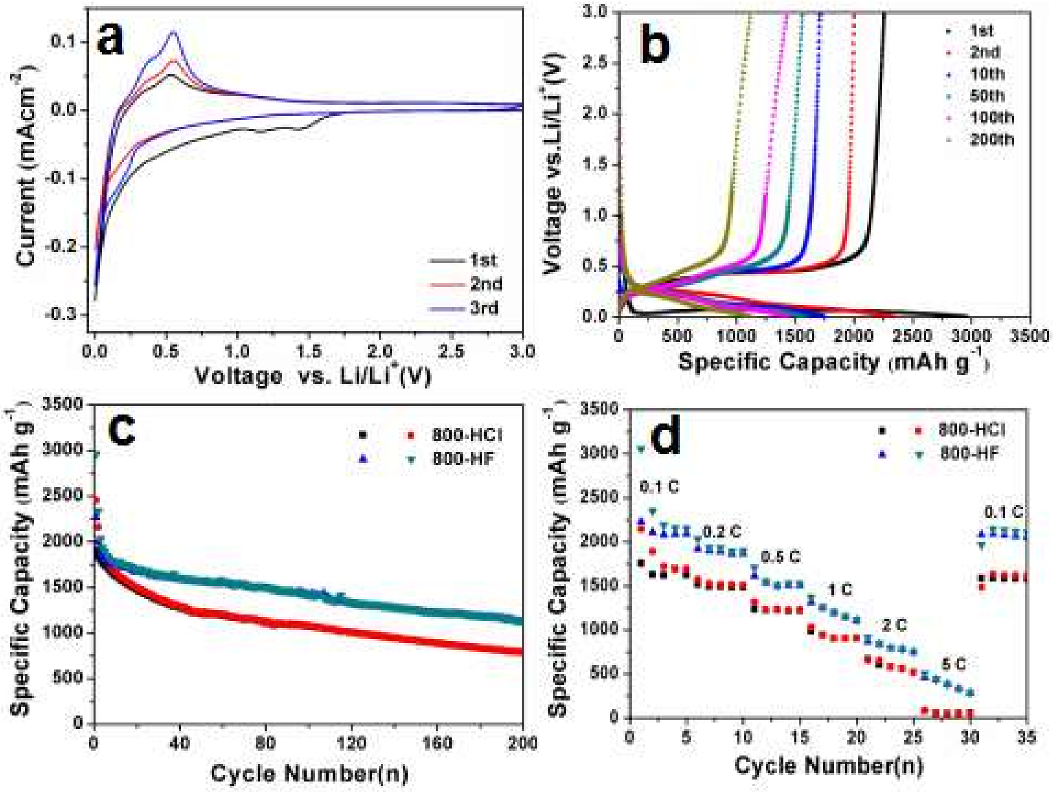

Figure S2. Cyclic voltammetry curves (a, scanning rate:0.2 mV sec^{-1}), galvanostatic discharge/charge profiles(b), cycling performance (c, current density: 0.2 C) and rate performance (d) of the 800-HF sample.

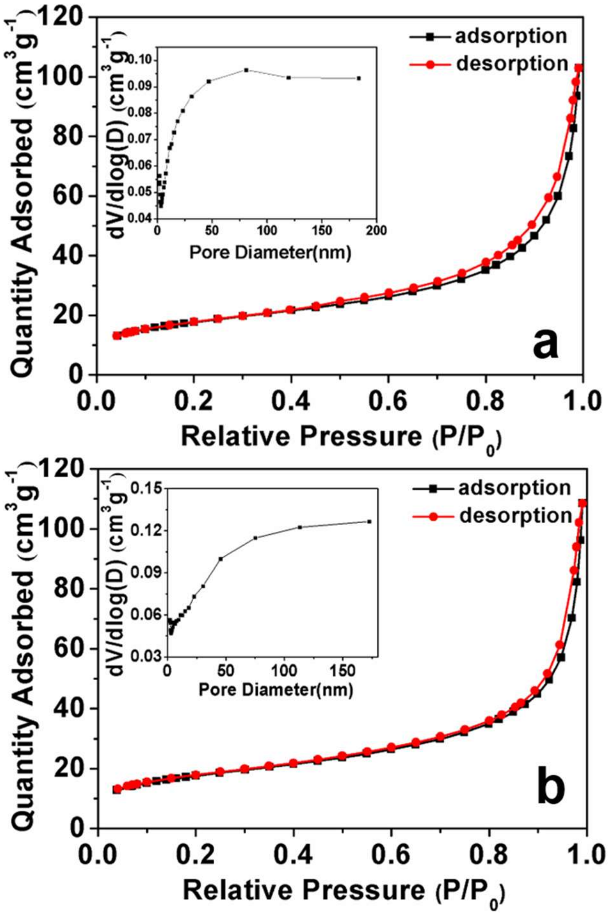

Figure S3. N_2 adsorption and desorption isotherms and the corresponding BJH pore size distribution curve of the 900-HCl sample(a) and 900-HF sample(b).

Table S1. Comparison of the cyclic performance of silicon based anodes in LIBs

sample	Current density/Ag^{-1}	Reversible capacity /$mAhg^{-1}$	Cylces	Capacity retention /%	Mass Loading /$mgcm^{-2}$	ref
silicon pomegranates	0.23	950	100	94	**3.12**	[28]
watermelon-inspired Si/C microspheres	0.3	620	500	80	**4.1**	[41]
Si/C composite	0.42	626	100	67	**5.0**	[42]
porous Si/C	0.05	1036	100	71	**3.0**	[43]
Non-filling carbon coating of porous silicon	0.1	1420	100	88	**2.0**	[44]
Nano -Si secondary clusters	0.25	1150	100	66	**2.0**	[45]
Si@rGO	0.5	1325	100	71	**2.6**	[46]
macro-/mesoporous Si	0.2	1298	100	76*	**2.4**	Our work

Note: *The capacity retention is calculated against the maximum capacity value after the activation process and corresponds to the number of cycles listed in the fourth column.

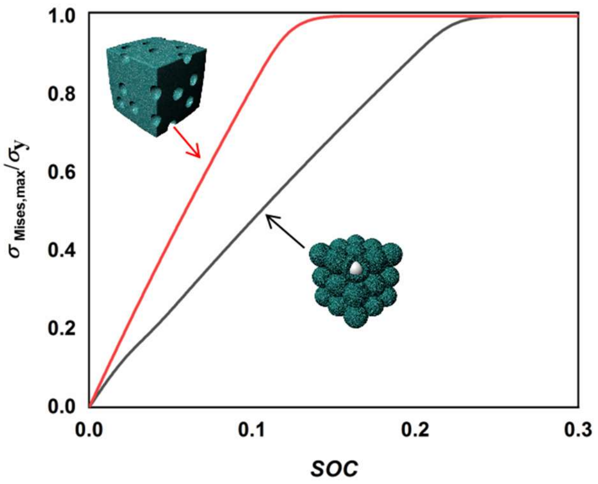

Figure S4. Evolution of peak Mises stress of silicon in spherical-shell structure of 700-HCl and porous structure of 800-HCl/900-HCl with *SOC*.
